# *Staphylococcus massiliensis* isolated from human blood cultures, Germany, 2017–2020

**DOI:** 10.1007/s10096-022-04409-4

**Published:** 2022-01-26

**Authors:** Katharina Last, Philipp M. Lepper, Philipp Jung, Hans-Joachim Schäfers, Sébastien Boutin, Klaus Heeg, Sören L. Becker, Dennis Nurjadi, Cihan Papan

**Affiliations:** 1 Center for Infectious Diseases, Institute of Medical Microbiology and Hygiene, Saarland University, Kirrberger Strasse, Building 43, 66421 Homburg, Germany; 2 Department of Pneumology, Allergology and Critical Care Medicine, Saarland University, Homburg, Germany; 3 Department of Thoracic and Cardiovascular Surgery, Saarland University, Homburg, Germany; 4 Department of Infectious Diseases, Medical Microbiology and Hospital Hygiene, University Hospital Heidelberg, Heidelberg, Germany

**Keywords:** Coagulase-negative staphylococci, *Staphylococcus massiliensis*, Non-model organisms, Novel species, Whole-genome sequencing, 16S rRNA, MALDI-TOF MS

## Abstract

Clinical and laboratory data on newly described staphylococcal species is rare, which hampers decision-making when such pathogens are detected in clinical specimens. Here, we describe *Staphylococcus massiliensis* detected in three patients at a university hospital in southwest Germany. We report the discrepancy of microbiological findings between matrix-assisted laser desorption/ionization time-of-flight mass spectrometry, 16S-rRNA polymerase chain reaction, and whole-genome sequencing for all three isolates. Our findings highlight the diagnostic pitfalls pertinent to novel and non-model organisms in daily microbiological practice, in whom the correct identification is dependent on database accuracy.

## Introduction

Coagulase-negative staphylococci (CoNS) are abundant inhabitants of the human skin and the mucosa with limited pathogenicity [1, 2], as opposed to *Staphylococcus aureus* [3] and other members of the *S*. *aureus* complex [4, 5]. Device-related infections are one of the most common manifestations of CoNS infections, causing high morbidity and mortality [6, 7]. With the advent of modern tools in routine microbiological diagnostics such as matrix-assisted laser desorption/ionization time-of-flight mass spectrometry (MALDI-TOF MS), pathogen detection has experienced a remarkable refinement within the past decade [8, 9]. However, reliable species identification by MALDI-TOF MS relies greatly on the quality and accuracy of the employed database. Over the past years, the species identification and differentiation of clinical CoNS isolates have improved significantly, leading to the identification of novel or “rare” species in clinical specimens [10]. Indeed, the “true” clinical relevance of many CoNS species is frequently underappreciated. *Staphylococcus massiliensis* is a recently described CoNS first reported in 2010 from a cerebral abscess [11], and later from healthy human skin [12]. In 2012, Zong postulated to conceptualize *S*. *massiliensis* as part of the normal human skin microflora [13]. Until today, data pertaining to the pathogenic potential of this microorganism and possible treatment approaches is scarce. Here, we report the challenges and limitations in the identification of *S. massiliensis* in blood cultures from three patients at a university hospital in southwest Germany.

## Methods

### Microbiological characterization and MALDI-TOF MS species identification

We performed a retrospective search of our microbiological database to identify *S. massiliensis* isolates in blood cultures from January 2017 to December 2020. We screened the database for blood culture isolates deposited as “coagulase-negative staphylococci,” suggesting that a definitive identification with MALDI-TOF MS failed in these isolates. All samples were processed in accordance with standard microbiological procedures, as described previously [4]. For isolate identification, a combination of MALDI-TOF MS (Biotyper™; Bruker Daltonics, Bremen, Germany, with the MBT Compass 4.1 database containing 7588 bacterial pathogens but not including *S. massiliensis*) according to the manufacturer’s protocols and sequencing of the 16S-rRNA coding gene was utilized (see below). Antimicrobial susceptibility testing (AST) was carried out on a VITEK II (BioMérieux, Marcy l’Étoile, France) and interpreted in accordance with the guidelines of the European Committee on Antimicrobial Susceptibility Testing (EUCAST, version 10.0). Data on the number of positive blood culture bottles and the time to positivity was collected.

### Sequencing of the 16S rRNA coding gene

A fragment of the 16S-rRNA coding gene was amplified, using the primer sequences (5′–3′) AGAGTTTGATCMTGGCTCAG (forward) and CCGTCAATTCMTTTGAGTTT (reverse), respectively. Subsequently, the PCR product was processed with the AMPure PCR purification kit (BeckmanCoulter, Krefeld, Germany) and dye-labeled using the DTCS Quick Start Kit (Sciex, Framingham, USA) with the primer sequences (5′–3′) AGAGTTTGATCMTGGCTCAG (forward) and GWATTACCGCGGCKGCTG (reverse), respectively. The dye-labeled products were purified with the CleanSEQ Dye-Terminator Removal Kit (BeckmanCoulter, Krefeld, Germany) and sequenced via capillary electrophoresis (GenomeLab GeXP; BeckmanCoulter, Krefeld, Germany). The forward and reverse strands were processed and analyzed individually. Sequencing and all kit reactions were performed according to the manufacturer’s protocols. The PCR protocols are displayed in Table 1.

**Table 1:** PCR protocols for amplification of the 16S rRNA coding gene and dye-labeling prior DNA sequencing

Amplification protocol (16S rRNA coding gene) 30 cycles			DCTS dye-labeling protocol prior sequencing 30 cycles		
Denaturing	95 °C	10 min (initial)	Denaturing	96 °C	10 min (initial)
Denaturing	95 °C	20 s (cycle)	Denaturing	96 °C	20 s (cycle)
Annealing	55 °C	60 s (cycle)	Annealing	50 °C	20 s (cycle)
Elongation	72 °C	3 min (cycle)	Elongation	60 °C	4 min (cycle)
Elongation	72 °C	10 min (final)	Elongation	60 °C	10 min (final)

### Whole-genome sequencing

DNA extraction and short-read Illumina sequencing were performed as previously described [14]. For correct species designation, core genome alignment was performed with publicly available *Staphylococcus haemolyticus* and *Staphylococcus saprophyticus* genomes, which showed the closest phylogenetic sequence homologies. The sequences were deposited at the NCBI GenBank under the Bioproject PRJNA750592.

### Patient characteristics

Review of the patient charts was performed to extract clinical data, including basic demographics, comorbidities, concomitant infectious syndromes and pathogens detected, presence of foreign bodies, and the antibiotic treatment.

### Ethics

This study was approved by the local ethics committee (Number 147/21). Due to the retrospective nature of the study and the anonymization of patient data, the need for informed consents was waived.

## Results

### Microbiological characterization

Between January 2017 and December 2020, a total of 203 isolates were found to be deposited as “coagulase-negative staphylococci” in our microbiological database. Of these, 181 were in a second step identified per MALDI-TOF MS as *Staphylococcus epidermidis* or other, common species, and in 13 cases, a polymicrobial culture was found and/or the initial suspicion of CoNS was refuted. In the remaining nine cases, MALDI-TOF MS yielded either *Staphylococcus* sp. or no reliable identification. Out of this cohort of nine isolates, four blood culture bottles from three different patients were positive for *S*. *massiliensis*. The clinical characteristics are provided in Table 2. MALDI-TOF MS yielded no reliable identification of all three isolates (all scores ≤ 1.47, for detailed information refer to Table 3), which were subsequently diagnosed as *S*. *massiliensis* via sequencing of the 16S-rRNA coding gene and further investigated by whole-genome sequencing (WGS). All isolates were methicillin-susceptible (Table 4).

**Table 2:** Characteristics of patients with positive blood cultures for *S*. *massiliensis*, treated at the Saarland University, Germany, 2017 to 2020

	Patient #1 (P1)	Patient #2 (P2)	Patient #3 (P3)
Age at presentation	67	79	53
Sex	Male	Female	Male
Number of positive blood culture bottle sets, in relation to total number of blood cultures drawn	1/1 (peripheral)	1/1 (peripheral)	1/2 (peripheral)
Positive bottle type(s)	Aerobic	Aerobic	Aerobic and anaerobic
Time to positivity of blood culture (hours)	28	73	18
Clinical syndrome	Aortic insufficiency, aortitis	Fever without source	COVID-19
Other pathogens recovered	*Staphylococcus epidermidis*, per culture (aortic valve) *Staphylococcus aureus*, per PCR (pSA-442) (aortic valve)	*Escherichia coli* (urine), *Citrobacter freundii* (urine)	-
Antibiotic treatment	Daptomycin; later switched to vancomycin + rifampicin	Ampicillin/sulbactam; meropenem	Azithromycin; piperacillin/tazobactam
Foreign body	Aortic valve and root replacement	Total knee replacement	Central venous catheter
Comorbidities	See above	Lung adenocarcinoma	COVID-19

**Table 3:** MALDI-TOF MS identification (ten highest scores) with ambiguous results, which were subsequently identified as *S. massiliensis* via sequencing of the 16S rRNA coding gene

Species	MALDI-TOF MS score	NCBI code
P1		
*Staphylococcus cohnii* spp. *cohnii*	1.38	74704
*Staphylococcus cohnii* spp. *cohnii*	1.37	74704
*Staphylococcus equorum* spp. *equorum*	1.37	246432
*Staphylococcus hominis*	1.35	1290
*Staphylococcus pasteuri*	1.34	45,972
*Staphylococcus haemolyticus*	1.34	1283
*Staphylococcus warneri*	1.31	1292
*Staphylococcus hominis* spp. *novobiosepticus*	1.31	145393
*Staphylococcus xylosus*	1.30	1288
*Lactobacillus mucosae*	1.29	97478
P2		
*Staphylococcus xylosus*	1.47	1288
*Staphylococcus equorum* spp. *equorum*	1.45	246432
*Staphylococcus cohnii* spp. *cohnii*	1.36	74704
*Staphylococcus saprophyticus* spp. *saprophyticus*	1.35	147452
*Staphylococcus cohnii* spp. *cohnii*	1.35	74704
*Staphylococcus xylosus*	1.30	1288
*Staphylococcus carnosus* spp. *utilis*	1.30	147,449
*Arthrobacter pascens*	1.29	1677
*Staphylococcus carnosus* spp. *carnosus*	1.28	147448
*Staphylococcus cohnii* spp. *urealyticus*	1.28	94138
P3		
*Staphylococcus carnosus* spp. *utilis*	1.45	147449
*Staphylococcus chromogenes*	1.44	46,126
*Staphylococcus lugdunensis*	1.39	28035
*Staphylococcus equorum* spp. *equorum*	1.35	246432
*Staphylococcus xylosus*	1.30	1288
*Staphylococcus xylosus*	1.30	1288
*Staphylococcus felis*	1.29	46127
*Staphylococcus xylosus*	1.28	1288
*Lactobacillus oligofermentas*	1.28	293371
*Staphylococcus warneri*	1.27	1292

**Table 4:** Antibiotic susceptibility profile of *S*. *massiliensis* isolates detected in blood cultures at the Saarland University, Germany, 2017 to 2020 (*n* = 3)

Antimicrobial substance	P1		P2		P3	
	MIC	Int	MIC	Int	MIC	Int
Flucloxacillin	≤ 0.25	S	≤ 0.25	S	≤ 0.25	S
Gentamicin	≤ 0.5	S	≤ 0.5	S	≤ 0.5	S
Ciprofloxacin	≤ 0.5	I	≤ 0.5	I	≤ 0.5	I
Moxifloxacin	≤ 0.25	S	≤ 0.25	S	≤ 0.25	S
Erythromycin	≤ 0.25	S	0.5	S	≥ 8	R
Clindamycin	≤ 0.25	S	≤ 0.25	S	≤ 0.25	S
Linezolid	1	S	1	S	2	S
Daptomycin	0.25	S	0.25	S	0.25	S
Vancomycin	≤ 0.5	S	≤ 0.5	S	≤ 0.5	S
Tetracycline	≤ 1	S	≤ 1	S	≤ 1	S
Tigecycline	≤ 1	S	≤ 0.12	S	≤ 0.12	S
Fosfomycin	≤ 0.12	R	≥ 128	R	≤ 8	S
Fusidic acid	≥ 128	R	2	R	≤ 0.5	S
Rifampicin	≤ 0.5	S	≤ 0.5	S	≤ 0.5	S
Co-trimoxazole	≤ 10	S	≤ 10	S	≤ 10	S

MICs are displayed as reported by VITEK2; antibiotic susceptibility was interpreted according to the EUCAST breakpoints v10.0. *I*, susceptible, increased exposure; *Int*, interpretation; *MIC*, minimal inhibitory concentration (in mg/L); *R*, resistant; *S*, susceptible

### Sequence analyses of the 16S-rRNA coding gene

PCR of isolate P1 (of patient #1) yielded a coverage of 100% each on the forward and reverse strands (671 and 465 base pairs), with 99.85% and 99.57% identification of *S*. *massiliensis*, respectively. Isolate P2 was diagnosed as *S*. *massiliensis* with coverage of 100% for both strands (forward 414 base pairs, reverse 386 base pairs), while identification resulted in 99.03% and 97.2%, respectively. Isolate P3 (forward 718 base pairs, reverse 424 base pairs) resulted in 100% coverage each, and an identification of 99.72% and 100%, respectively, as *S*. *massiliensis*.

### WGS

**Fig. 1:**
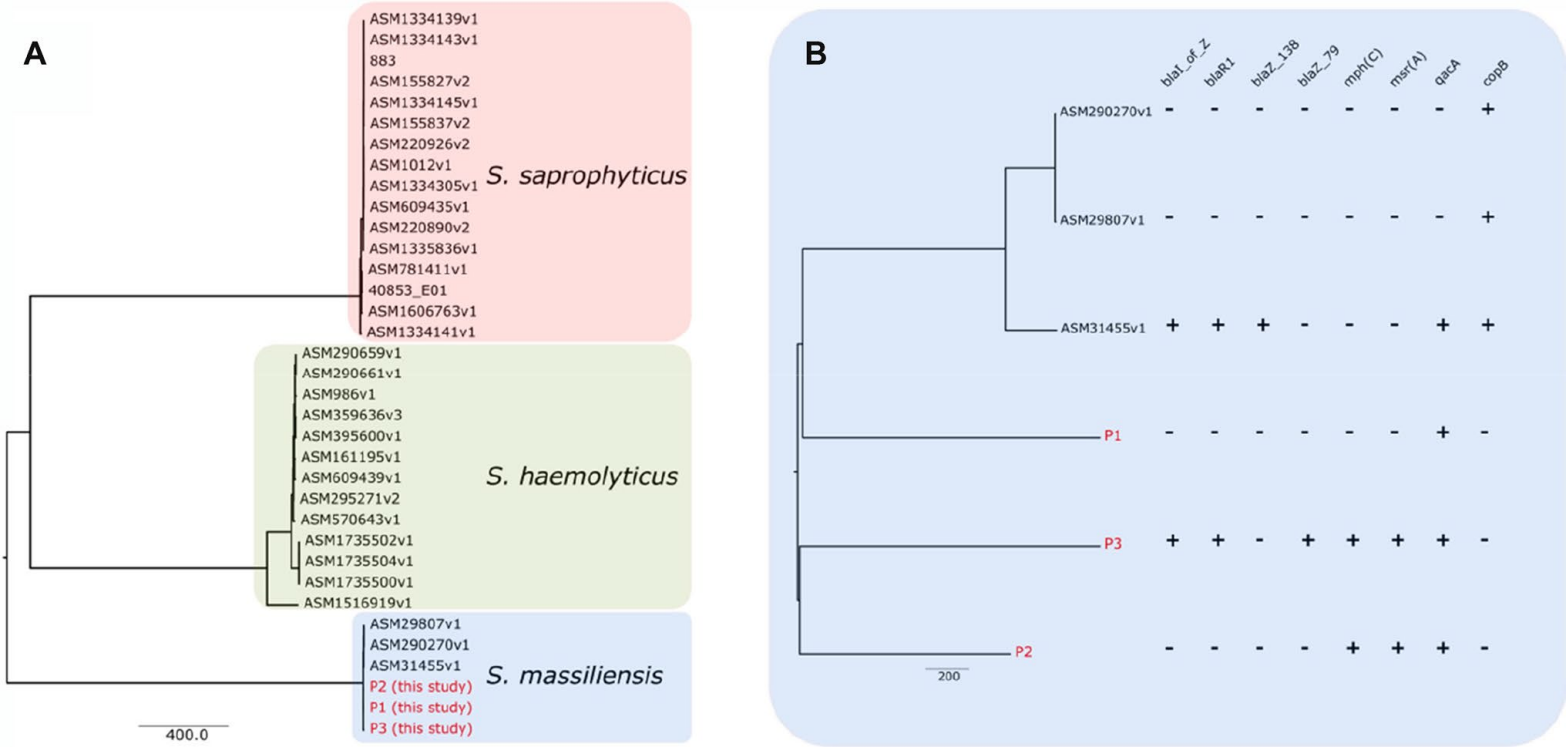
Phylogenetic tree of *Staphylococcus massiliensis* isolates and closely related *Staphylococcus species*. **A** Genetic identification of our isolates (P1, P2, and P3) as *S. massiliensis* compared to publicly available genomes of closely related species (*S. saphrophyticus*, *S. hemolyticus*, and *S. massiliensis*). **B** Phylogenetic tree based on the core genome of *S. massiliesis* (*n* = 6) from our study and those previously published. Presence of antimicrobial resistance genes was assessed using the RESfinder database

The draft genome was analyzed using KmerFinder and two different species were suggested as best hit: *S. haemolyticus* and *S. saprophyticus*. *S. massiliensis* is not included in the database of KmerFinder; therefore, we compared our sequences to the 3 publicly available genomes of *S. massiliensis* as well as the complete genome publicly available in the refseq database. The core genome used for the comparison was relatively small (27 genes, 1661 polymorphic sites) but revealed a close phylogentic relationship between our isolates and the 3 genomes belonging to *S. massiliensis* (Fig. 1A; Table 5). To validate these results, we refined the core genome (1987 genes, 3093 polymorphic sites) using only the common genes among those 6 isolates. This analysis validated the genetic identity of our isolate as *S. massiliensis* with a higher confidence. These results are also in line with the average nucleotide identity (ANI) of 99.29% (99.19–99.38%) between our 3 isolates and the 3 available genomes of *S. massiliensis*, with an ANI value > 95% being a reliable indicator for species identity [15]. Although our analyzed isolates all belonged to *S. massiliensis*, they showed a different resistome pattern (Fig. 1B). We found a high level of resistance with 6 resistance genes in isolate P3, while the reference genomes were overall more susceptible to antibiotics.

**Table 5:** Sequencing statistics

Patient	Isolate ID	Species	Accession	Coverage	Number of contigs	Largest contigs	Total length	GC (%)	N50	N75	L50	L75
P1	1253363	*Staphylococcus massiliensis*	SAMN20474413	37	19	1389804	2408521	36.51	1389804	648304	1	2
P2	1236816	*Staphylococcus massiliensis*	SAMN20474414	76	39	313148	2368708	36.52	143157	87495	6	11
P3	1239837	*Staphylococcus massiliensis*	SAMN20474412	32	56	292790	2379679	36.5	92517	48998	8	16

*Draft genomes are publicly available at the NCBI Gen*B*ank under the Bioproject number PRJNA750592*

## Discussion

We describe here the incongruency of bacterial species identification for *S. massiliensis* using three different methods, namely sequencing of the 16S rRNA coding gene, MALDI-TOF MS, and WGS. While Sanger sequencing of the 16S rRNA coding gene correctly identified *S. massiliensis*, both mass spectrometry and next-generation sequencing were unable to clearly distinguish *S. massiliensis* from closely related staphylococcal species. Indeed, Zong previously reported the misidentification as *Staphylococcus simulans* by the MicroScan Walkaway 96 system [13]. Our results demonstrate that even modern and high-resolution identification techniques, such as MALDI-TOF MS and WGS, are dependent on the quality and currentness of the database. Although WGS is frequently regarded as a new gold standard for the molecular characterization of bacteria, the used databases are not necessarily reliable for newly described species and non-model organisms. The clinical relevance of *S. massiliensis* is still unclear. In all three patients, we detected *S. massiliensis* in the blood culture. Despite clear clinical signs of infection and inflammatory processes, the interpretation of blood culture positivity for *S. massiliensis* remains difficult. In both, patients 1 and 2, other clinically relevant pathogens were detected, and COVID-19 was diagnosed in patient 3, thus rendering a causative role of *S. massiliensis* uncertain. In addition, only one blood culture pair was taken for patients 1 and 2, and the time to positivity for all blood culture bottles was rather long (> 16 h) for all three patients, so that contamination cannot be completely ruled out. Thus, no definitive statement can be made regarding the clinical relevance of the *S*. *massiliensis* findings in our patients. Nonetheless, the accuracy of species identification is essential for further investigations on the clinical relevance of *S. massiliensis* as a potential pathogen. While the detection of CoNS in blood cultures is frequently considered contamination, specific CoNS species, such as *Staphylococcus epidermidis* and *Staphylococcus capitis*, may well be clinically relevant, in particular if indwelling devices are present [16]. The first description of *S*. *massiliensis* dates back to 2010, when Al Malsalma and colleagues reported the detection of this staphylococcal species from clinical specimen of a 52-year-old male patient (from 2005) with a brain tumor [11]. He developed a cerebral abscess after neurosurgery, which was drained and sent for microbiological diagnostics. Indeed, CoNS can cause post-surgical intracranial infections, rendering the findings of Al Malsalma et al. plausible. However, *S. massiliensis* has also been suggested as commensal of the human skin as it has been found in the skin of a fresh wound and healthy human skin [12, 13]. On a different note, consistent with the findings of Zong et al. of the presence of a mobile staphylococcal cassette chromosome *mec* in *S. massiliensis* [17], we detected the presence of multiple resistance genes in one of our isolates. The presence of clinically relevant antimicrobial resistance genes in skin commensals may pose therapeutic challenges and may be relevant for the acquisition of antibiotic resistances in facultative pathogens [18], such as members of the *S. aureus* complex, which warrants further investigations.

In conclusion, our data add to the scarce body of evidence that *S*. *massiliensis* may be detected in clinical samples for routine microbiological diagnostics. Thus far, the clinical relevance is still unclear and further clinical and experimental studies are needed to elucidate the pathogenic properties and virulence of *S. massiliensis*. The technical advancement in microbiological diagnostics will introduce new depths in species identification and specification of novel organisms within the next years.

## Data Availability

All relevant data are published within the article. Genome sequences of the 3 isolates investigated are available at the NCBI GenBank under project number PRJNA750592.
